# The complete chloroplast genome of *Caryota obtusa*, an endangered and economically important species

**DOI:** 10.1080/23802359.2020.1768944

**Published:** 2020-05-27

**Authors:** Yongqian Gao, Mei Lv, Tao Cui, Xiaoli Wan

**Affiliations:** a Yunnan Forestry Technological College, Kunming, PR China; b Forest Pest Quarantine and Control Station, Forestry Administration of Weining County, Weining, PR China; c Lincang Academy of Forestry Sciences, Lincang, PR China

**Keywords:** Chloroplast complete genome, *Caryota obtusa*, Arecaceae, phylogenetic analysis

## Abstract

*Caryota obtusa* is an endangered and economically important species of the Arecaceae. The complete chloroplast genome sequence of this species is a circular molecule of 159,882 bp in size, including a pair of inverted repeats with length of 27,271 bp, separated by a large single-copy (87,645 bp) region and a small single-copy region (17,695 bp). In total, there are 131 genes, encoding 79 protein-coding genes, 40 tRNAs, and 10 rRNA genes, in which 123 genes, 69 CDSs, 37 tRNAs, and 10 rRNAs are unique, respectively. Phylogenetic inference confirmed the monophyly of the *Caryota* genus and its delimitation in subfamily Coryphoideae.

*Caryota obtusa* Griffith is a species of flowering plant in the palm family (Arecaceae) distributed in montane rain forests, usually on limestone soils of south Yunnan Province of China, and also in India, Laos, Myanmar, Thailand, and Vietnam (Pei et al. 2010). The plant has important economic value, widely cultivated in gardens in the tropics for ornamental purposes, and used as a natural fiber and a substitute for the starch in sago (Kumai and Rajyalakshmi 2000; Palanikumar and Subbiah 2019). Habitats of this species are constantly losing because of deforestation, and it is one of the national grade-2 key protected wild plants of China. To facilitate taxonomy and phylogenetic studies, as well as conservation of this species, we sequenced and assembled the complete chloroplast genome of it, which has not been reported before.

Genomic DNA was extracted from fresh leaves of *C. obtuse* using a modified CTAB method (Porebski et al. 1997). Fresh leaves were collected from Kunming Botanic Garden, Yunnan Province, China (25°08′ N, 102°44′ E, 1918 m a.s.l.), and the voucher specimen was deposited in the Herbarium of Kunming Institute of Botany (specimen number: GYQ-17001). After DNA extraction, fragmentation and short-insert libraries (300 bp) construction were performed using the procedure of manufacture’s protocol (Illumina Inc., USA), and the paired-end sequencing was performed on an Illumina Hiseq X-Ten sequencer. The clean reads (about 31.5 million) were assembled using the program NOVOPlasty v3.7.1 (Dierckxsens et al. 2017) with complete chloroplast genome of a congenic species, *Coryota mitis* (GenBank accession No.: NC022948). The assembled chloroplast genome was annotated using GeSeq (Tillich et al. 2017).

The complete chloroplast genome of *C. obtuse* was assembled to 159,882 bp in size, with an average coverage of 332, and GC content of 37% (GenBank accession MT409429). The chloroplast genome consists of a pair of inverted repeats with length of 27,271 bp, separated by a large single-copy (87,645 bp) region and a small single-copy region (17,695 bp). In total, it contains 131 genes, encoding 79 protein-coding genes, 40 tRNAs, and 10 rRNA genes, in which 123 genes, 69 CDSs, 37 tRNAs, and 10 rRNAs are unique, respectively.

We further conducted a phylogenetic inference to determine the relationship of *C. obtusa* with its close related species, which belong to subfamily Coryphoideae, including 12 *Coryphoid* species with complete chloroplast genome downloaded from GenBank. The whole chloroplast genome sequences of these 13 species were aligned using MAFFT v7.450 (Katoh and Standley 2016), and the maximum-likelihood tree was then constructed using RaxML v8.0.0 under the GTR CAT model (Stamatakis 2006), branch supports were tested using 500 bootstraps replicates. The phylogenetic tree shown that the two species of *Caryota* analyzed in this study formed a monophyly clade with bootstrap value of 100%, and *Caryota* genus was sister to a clade consist of species from genera *Arenga* and *Wallichia*, indicating close relationships between these genera. Our result also confirms the delimitation of genus *Caryota* in subfamily Coryphoideae. These findings are generally consistent with former studies (Asmussen et al. 2006; Baker et al. 2009) (Figure 1).

**Figure 1.**
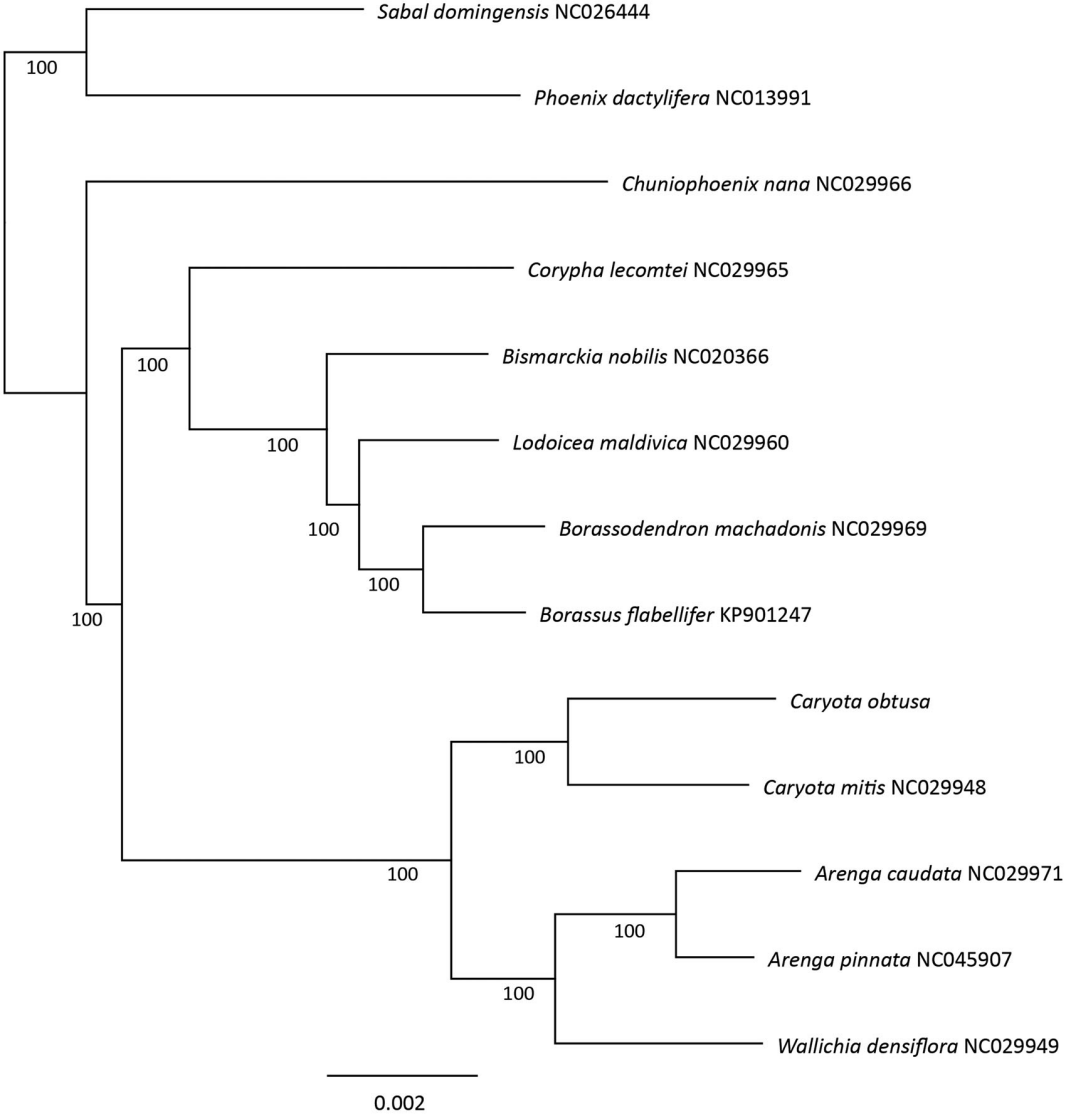
The maximum likelihood tree of *Caryota obtusa* and 12 Arecaceae species based on complete chloroplast genome. Bootstrap support values were indicated bellowed the branches.

## Data Availability

The data that support the findings of this study are available in GenBank at https://www.ncbi.nlm.nih.gov/genbank/, reference number [MT409429].
